# Supplementary figures and images for: Integrative study of skeletal muscle mitochondrial dysfunction in a murine pancreatic cancer-induced cachexia model
Source: eLife. 2024 Oct 18;13:RP93312. doi: 10.7554/eLife.93312 (PMC11488855; doi:10.7554/eLife.93312)

Uncropped blot with the relevant band and samples clearly labelled

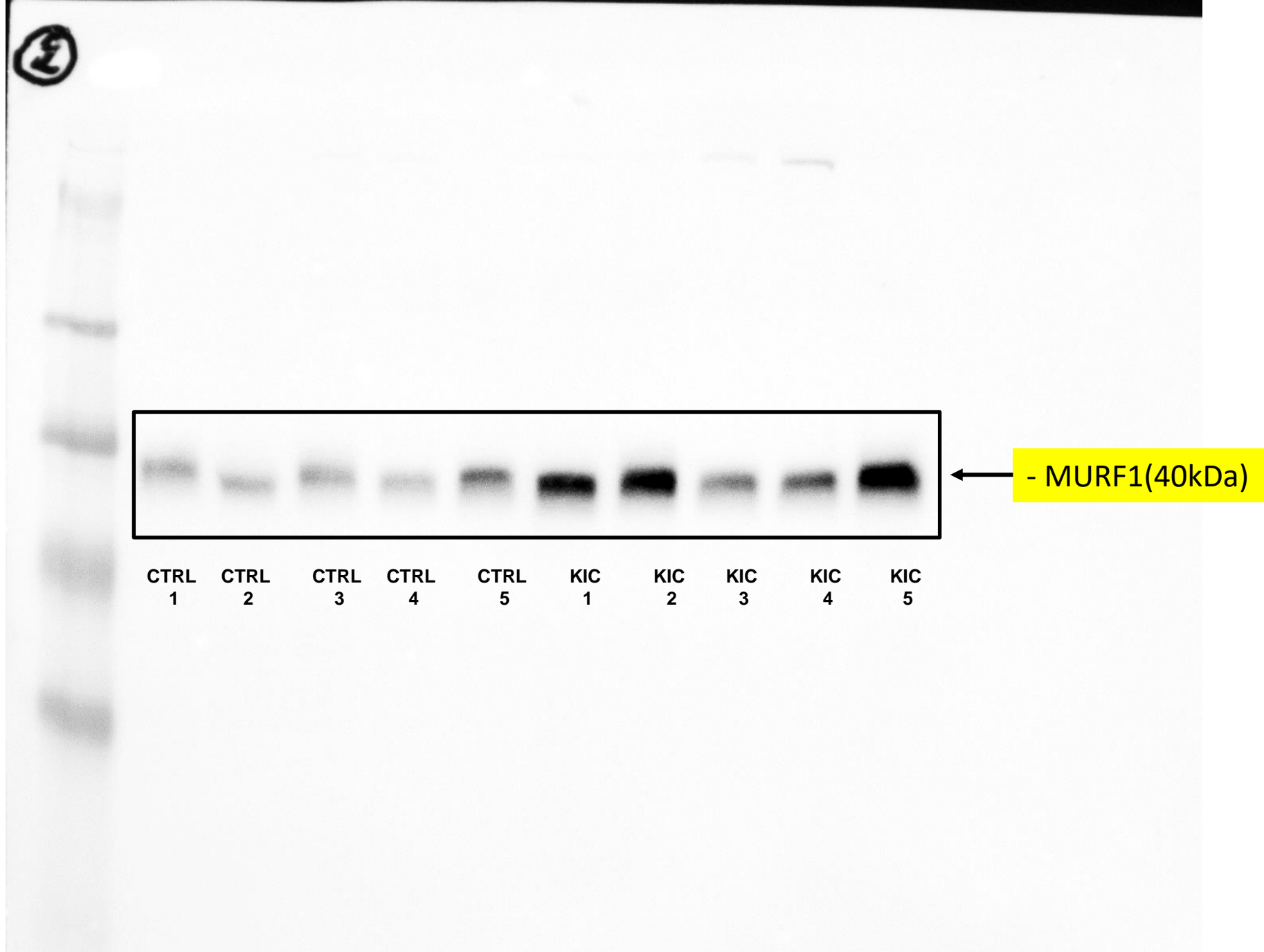

Supplement: Figure 2—source data 1. [file elife-93312-fig2-data1.zip › eLife-93312-Fig2-data1/Figure 2 - Source data 1 uncropped and labelled gels for Figure 2.pdf]

Uncropped gel with the relevant band and samples clearly labelled

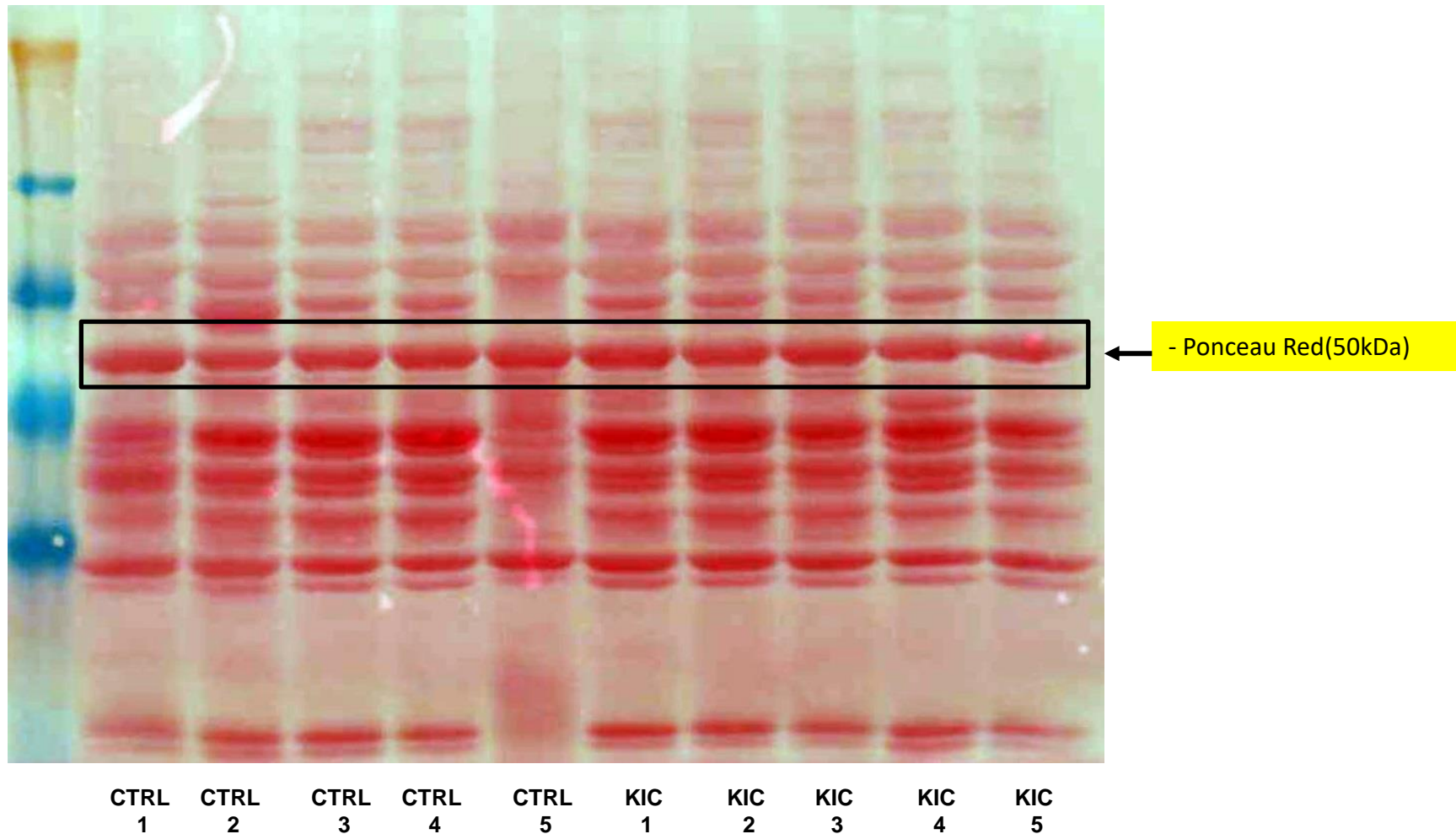

Supplement: Figure 2—source data 1. [file elife-93312-fig2-data1.zip › eLife-93312-Fig2-data1/Figure 2 - Source data 2 uncropped and labelled gels for Figure 2.pdf]

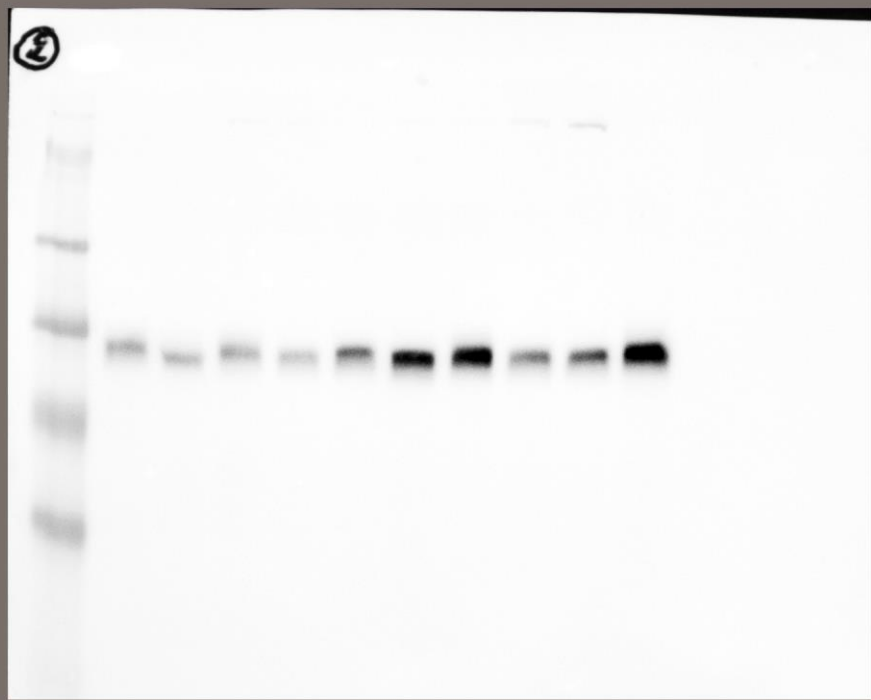

Supplement: Figure 2—source data 2. [file elife-93312-fig2-data2.zip › eLife-93312-Fig2-data2/Figure 2 - Source data 1 raw unedited gels for Figure 2.pdf]

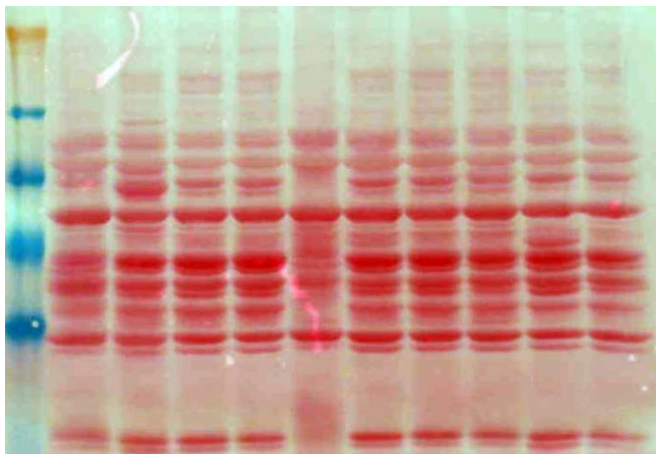

Supplement: Figure 2—source data 2. [file elife-93312-fig2-data2.zip › eLife-93312-Fig2-data2/Figure 2 - Source data 2 raw unedited gels for Figure 2.pdf]

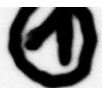

## Mitochondrial complexes – Western blot

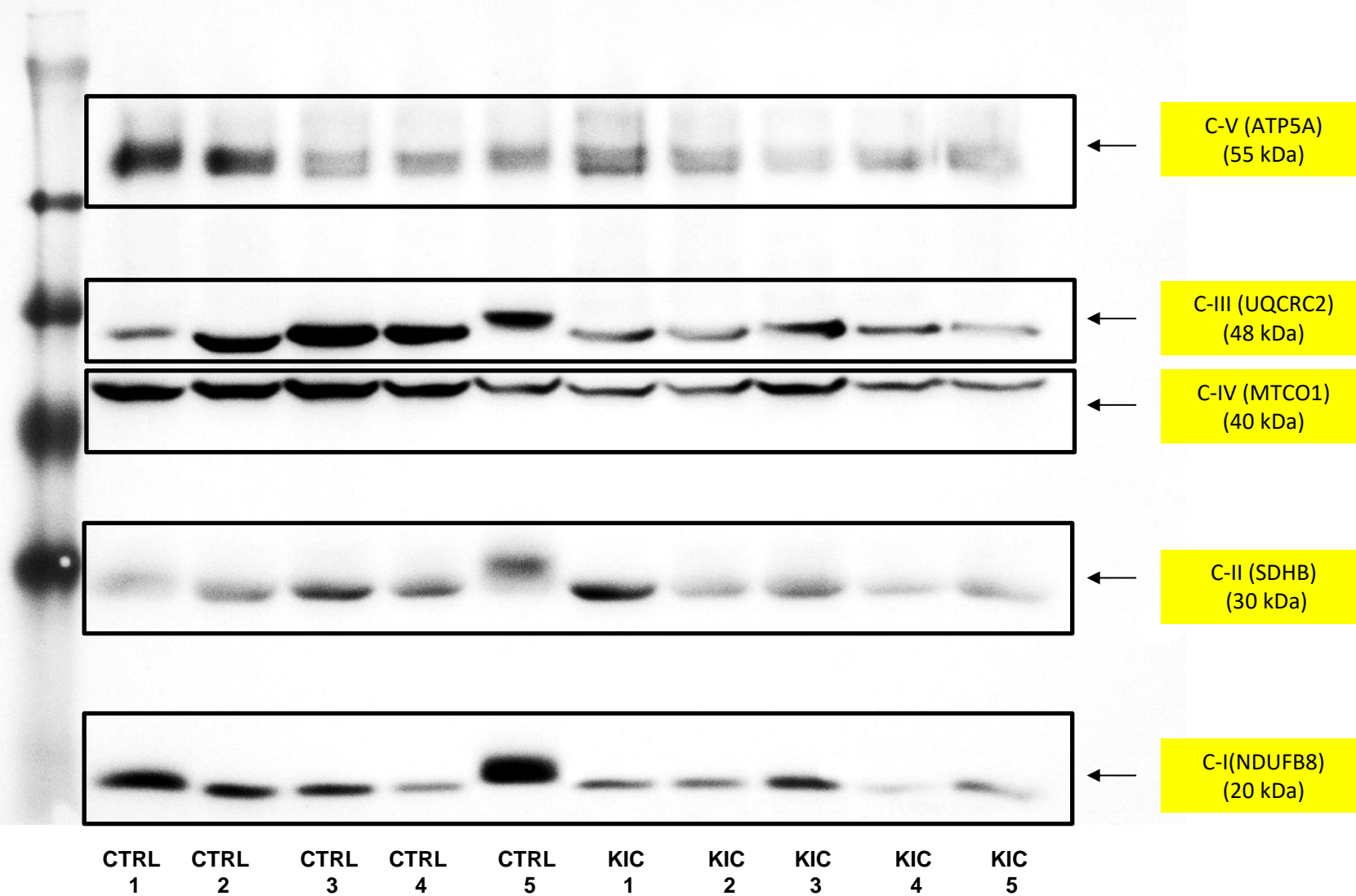

Supplement: Figure 4—source data 1. [file elife-93312-fig4-data1.zip › eLife-93312-Fig4-data1/Figure 4 - Source data 1 uncropped and labelled gels for Figure 4.pdf]

Mitochondrial complexes – Uncropped Ponceau Red

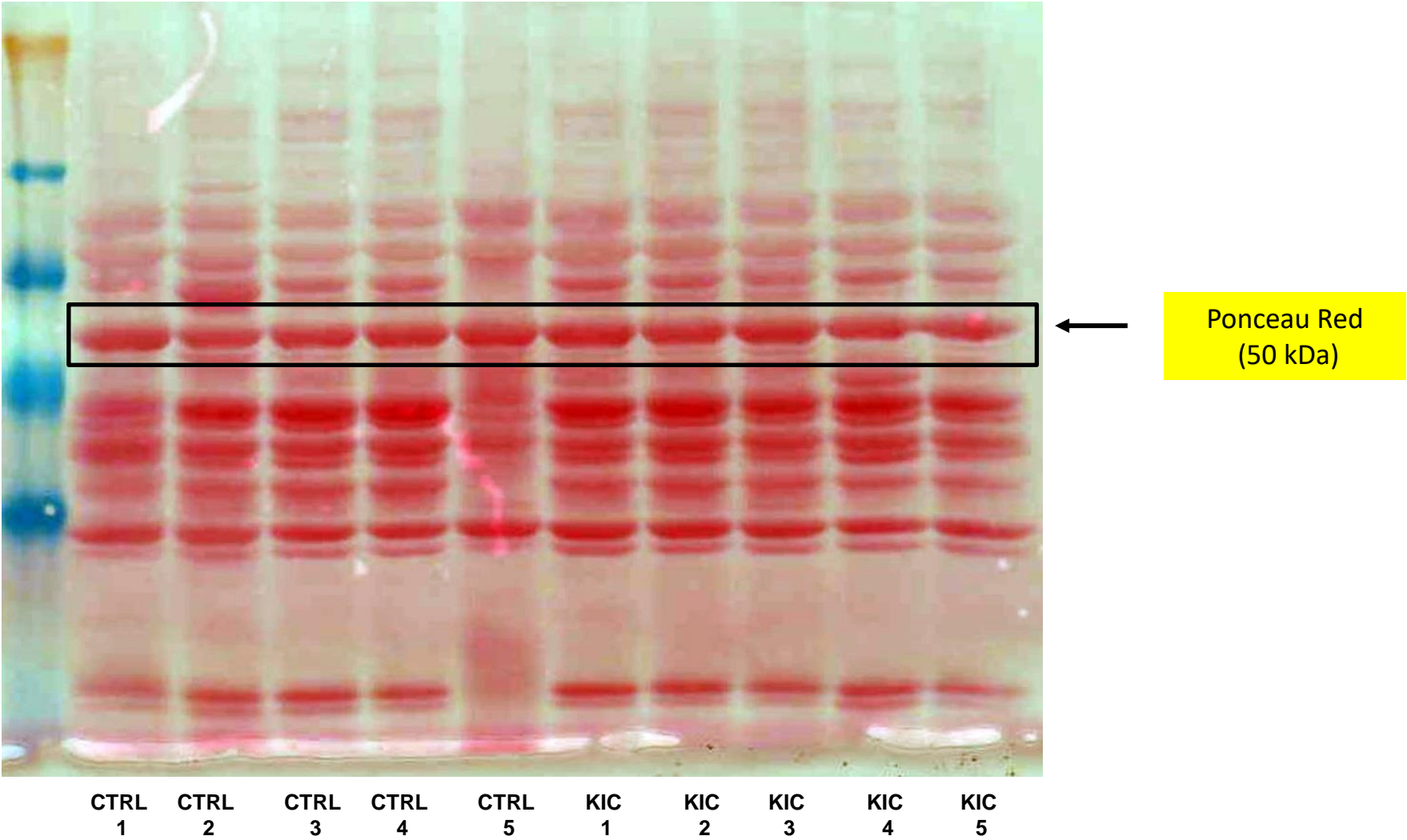

Supplement: Figure 4—source data 1. [file elife-93312-fig4-data1.zip › eLife-93312-Fig4-data1/Figure 4 - Source data 2 uncropped and labelled gels for Figure 4.pdf]

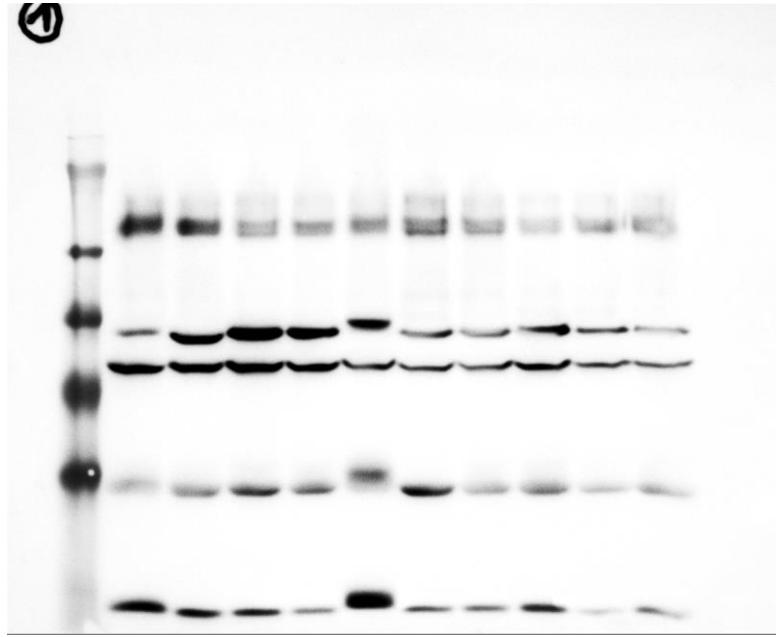

Supplement: Figure 4—source data 2. [file elife-93312-fig4-data2.zip › eLife-93312-Fig4-data2/Figure 4 - Source data 1 raw unedited gels for Figure 4.pdf]

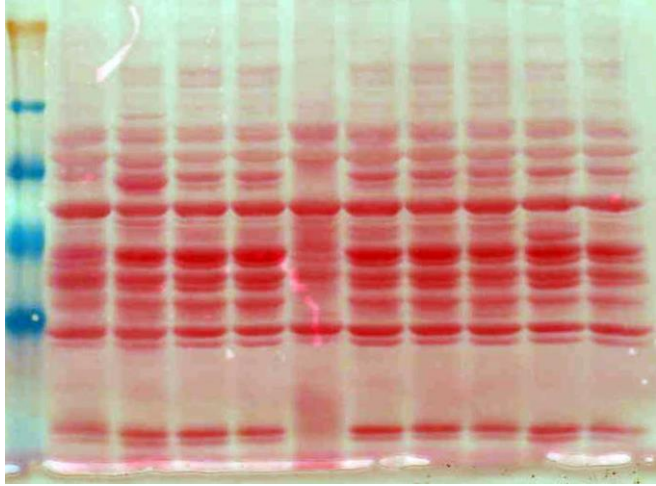

Supplement: Figure 4—source data 2. [file elife-93312-fig4-data2.zip › eLife-93312-Fig4-data2/Figure 4 - Source data 2 raw unedited gels for Figure 4.pdf]

## Mitofusin 1+2 staining

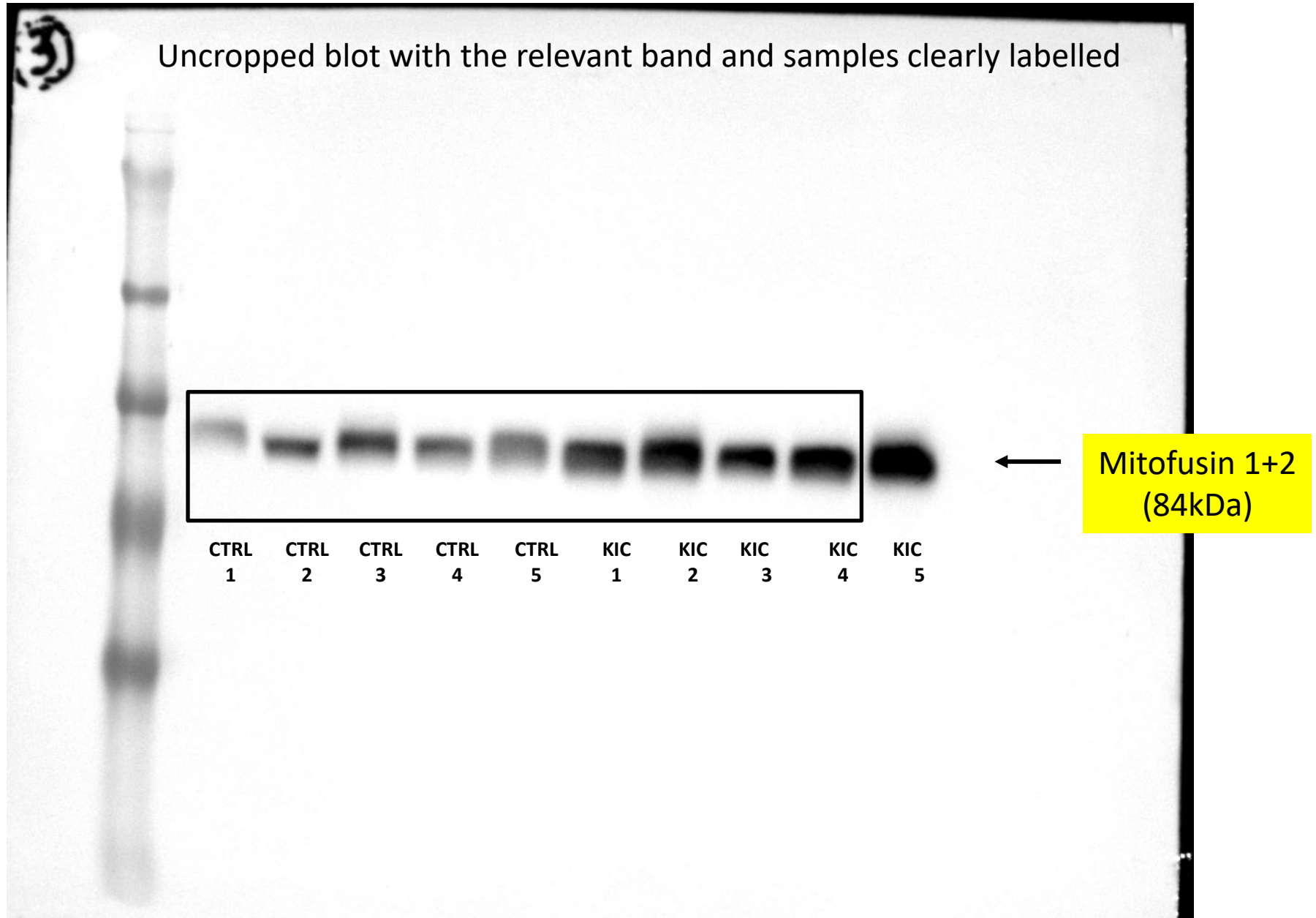

Supplement: Figure 5—source data 1. [file elife-93312-fig5-data1.zip › eLife-93312-Fig5-data1/Figure 5 - Source data 1 uncropped and labelled gels for Figure 5.pdf]

## OPA1 - staining

Uncropped blot with the relevant band and samples clearly labelled

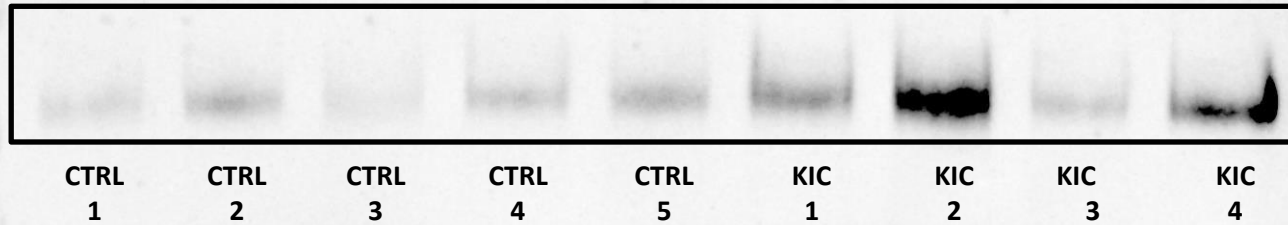

- OPA1  
(111kDa)

Supplement: Figure 5—source data 1. [file elife-93312-fig5-data1.zip › eLife-93312-Fig5-data1/Figure 5 - Source data 2 uncropped and labelled gels for Figure 5.pdf]

## PGC1a staining

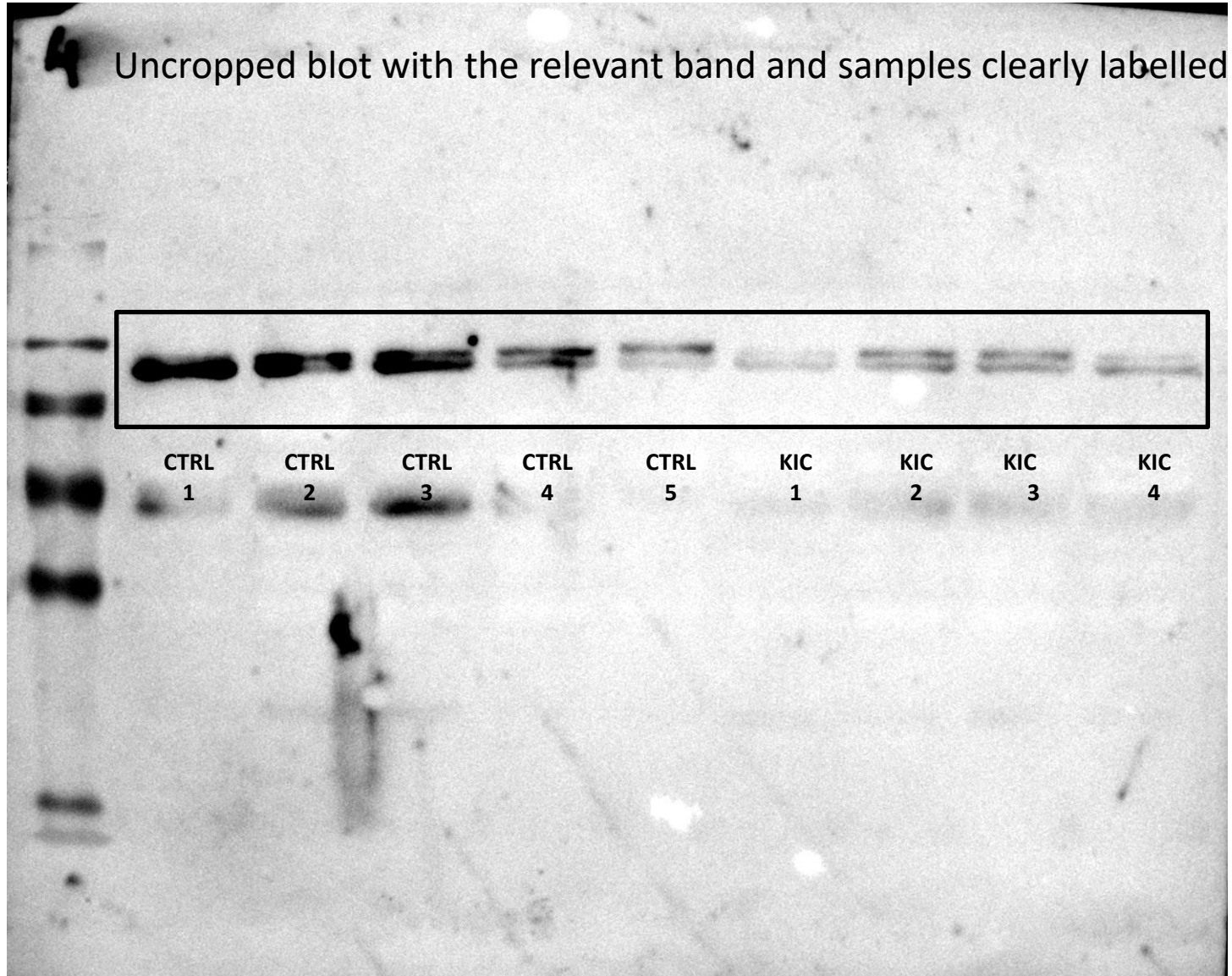

Supplement: Figure 5—source data 1. [file elife-93312-fig5-data1.zip › eLife-93312-Fig5-data1/Figure 5 - Source data 3 uncropped and labelled gels for Figure 5.pdf]

## TFAM staining

Uncropped blot with the relevant band and samples clearly labelled

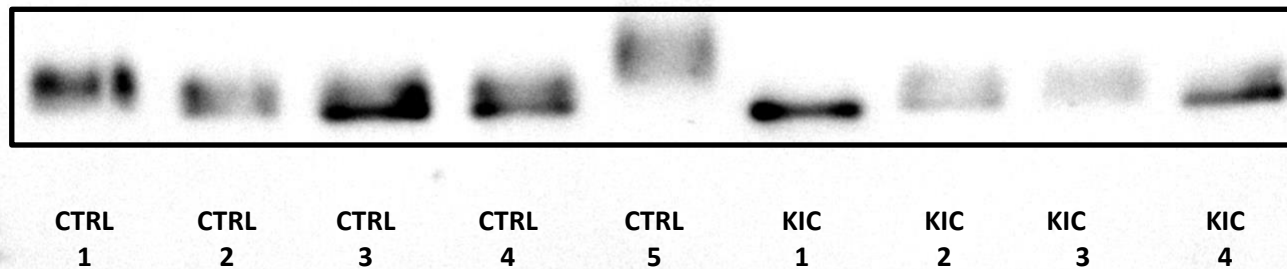

← - TFAM  
(29kDa)

Supplement: Figure 5—source data 1. [file elife-93312-fig5-data1.zip › eLife-93312-Fig5-data1/Figure 5 - Source data 4 uncropped and labelled gels for Figure 5.pdf]

## PGC1a – Amido Black

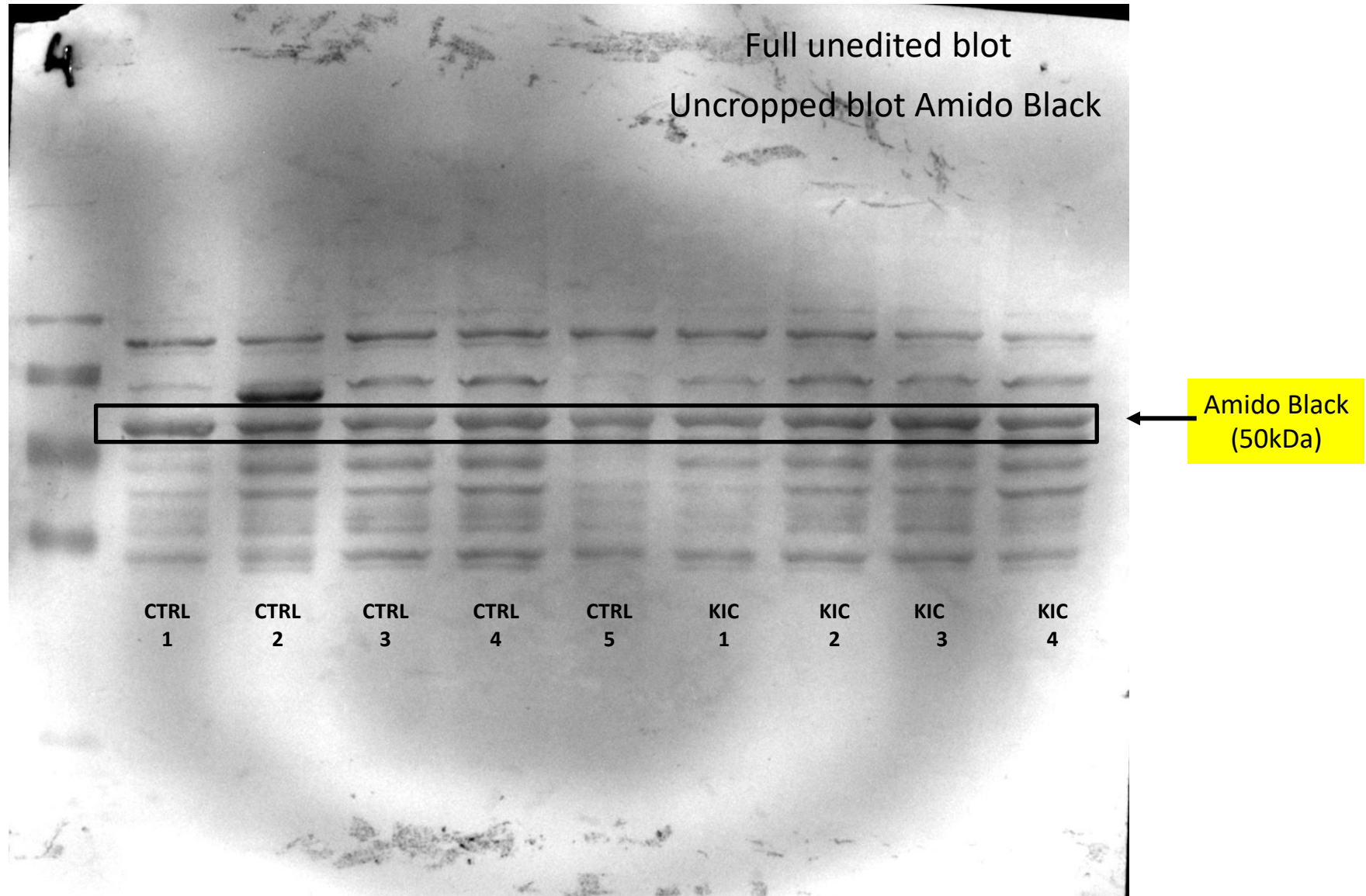

Supplement: Figure 5—source data 1. [file elife-93312-fig5-data1.zip › eLife-93312-Fig5-data1/Figure 5 - Source data 5 uncropped and labelled gels for Figure 5.pdf]

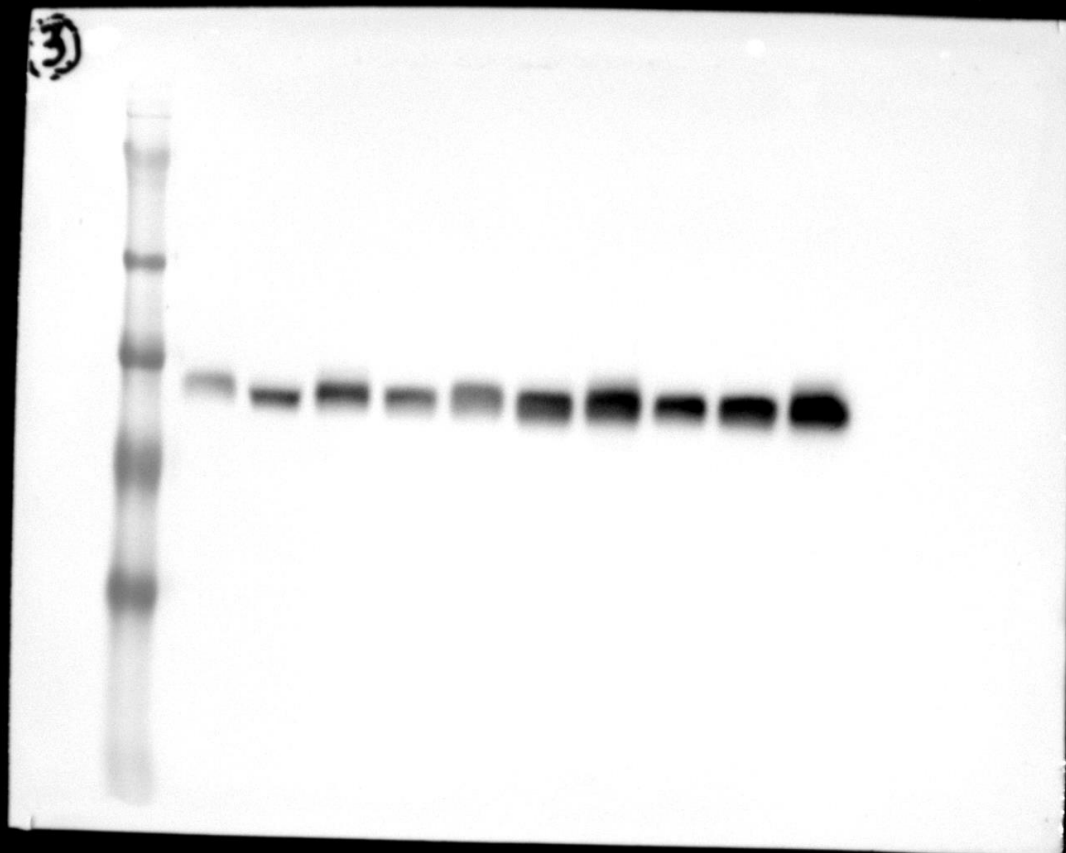

Supplement: Figure 5—source data 2. [file elife-93312-fig5-data2.zip › eLife-93312-Fig5-data2/Figure 5 - Source data 1 raw unedited gels for Figure 5.pdf]

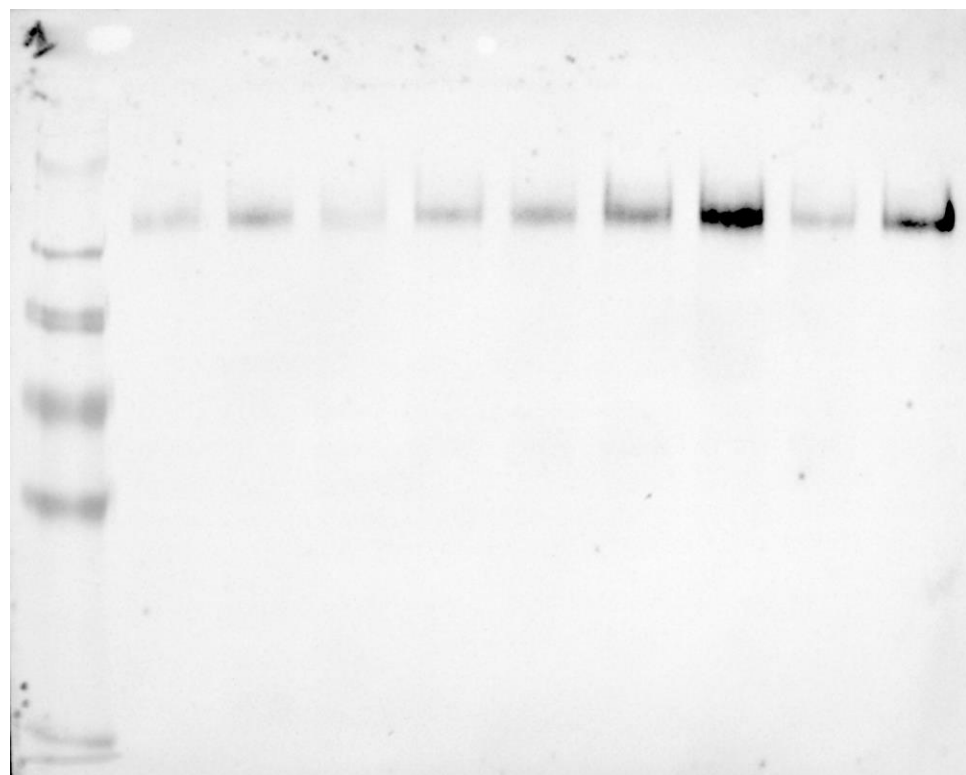

Supplement: Figure 5—source data 2. [file elife-93312-fig5-data2.zip › eLife-93312-Fig5-data2/Figure 5 - Source data 2 raw unedited gels for Figure 5.pdf]

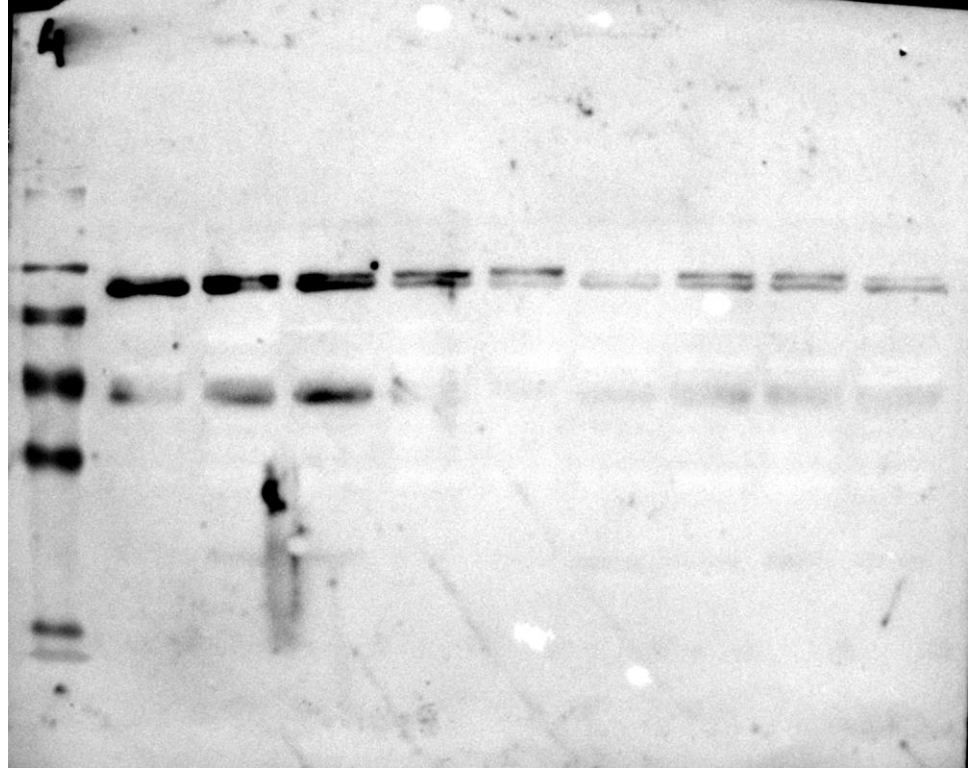

Supplement: Figure 5—source data 2. [file elife-93312-fig5-data2.zip › eLife-93312-Fig5-data2/Figure 5 - Source data 3 raw unedited gels for Figure 5.pdf]

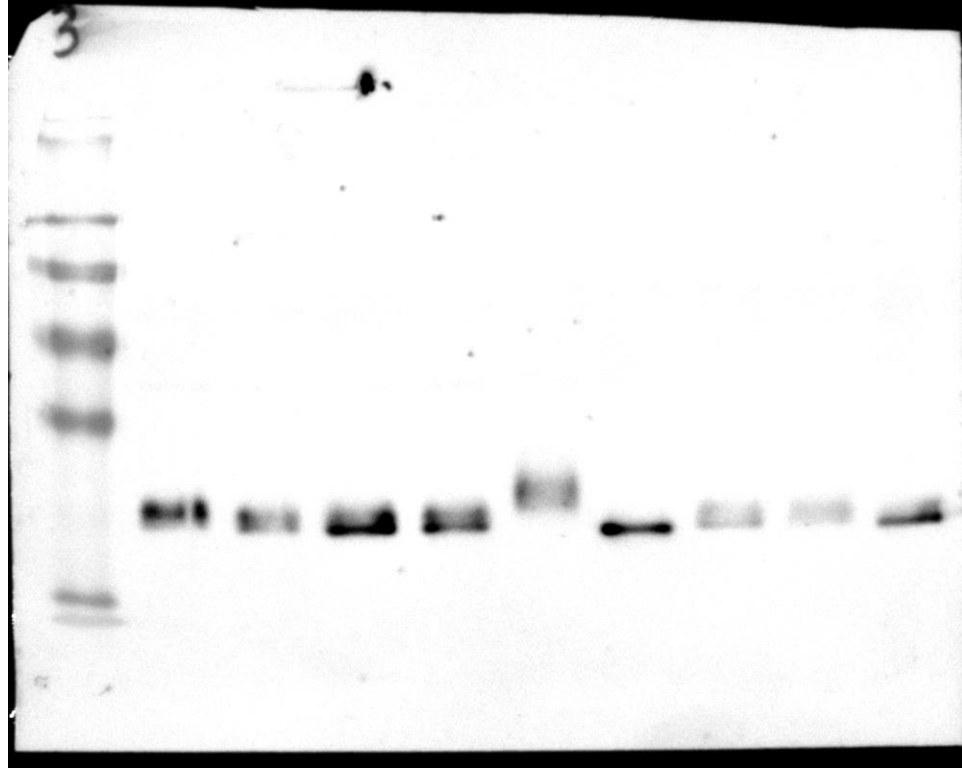

Supplement: Figure 5—source data 2. [file elife-93312-fig5-data2.zip › eLife-93312-Fig5-data2/Figure 5 - Source data 4 raw unedited gels for Figure 5.pdf]

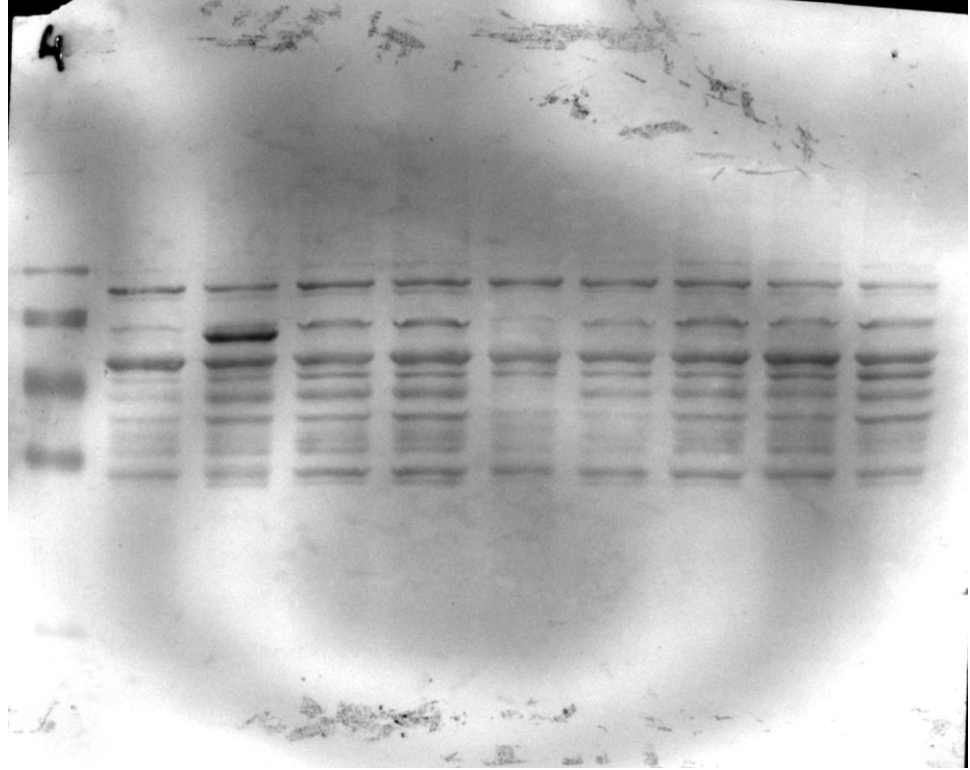

Supplement: Figure 5—source data 2. [file elife-93312-fig5-data2.zip › eLife-93312-Fig5-data2/Figure 5 - Source data 5 raw unedited gels for Figure 5.pdf]
